# Evaluation of the association between hyperuricemia and coronary artery disease: A STROBE-compliant article

**DOI:** 10.1097/MD.0000000000012926

**Published:** 2018-11-02

**Authors:** Ming Lan, Bing Liu, Qing He

**Affiliations:** Department of Cardiology, Beijing Hospital, Dongdan, Beijing, China.

**Keywords:** coronary angiography, female sex, prevalence and extent of CAD, severity of CAD, uric acid

## Abstract

The aim of the study was to assess the independent contribution of hyperuricemia to coronary artery disease (CAD) confirmed by coronary angiography (CAG), and to explore associations between serum uric acid (SUA) level and CAD.

We performed a retrospective cohort study of 5069 patients who underwent CAG. Patients were divided into groups: hyperuricemia (n = 1178) versus nonhyperuricemia (n = 3891) and CAD (n = 3433) versus non-CAD (n = 1636).

The incidence of CAD was higher in the hyperuricemia group than in the nonhyperuricemia group (71.5% vs 66.6%, *P* = .002). Hyperuricemia and CAD were significantly correlated in women (odds ratio = 1.509, 95% confidence interval, 1.106–2.057, *P* = .009). And there were trends, higher SUA quartiles were associated with higher percentage of CAD (62.3%, 68.0%, 68.9%, and 71.7% for quartiles 1, 2, 3, and 4, respectively, *P* < .001), and the incidence of 3-vessel disease increased (25.2%, 26.4%, 27.2%, and 31.1% for quartiles 1, 2, 3, and 4, respectively, *P* = .001) and that of normal vessel decreased (37.7%, 32.0%, 31.1%, and 28.3% for quartiles 1, 2, 3, and 4, respectively, *P* < .001) across quartiles, these trends were found in female group, but not in male group. The SUA level significantly increased as the number of diseased vessels increased (*P* < .001).

There was an independent correlation between hyperuricemia and CAD in women. A higher SUA level was associated with a higher incidence of 3-vessel disease in women.

## 1 Introduction

Hyperuricemia has been associated with coronary artery disease (CAD) since as far back as the 1850s, when Gertler^[1]^ first put forward the notion. Previous clinical investigations have demonstrated that there is a close relationship between hyperuricemia (or serum uric acid [SUA]) and CAD.^[2–10]^ However, whether hyperuricemia is an independent risk factor for CAD, the nature of the relationship between hyperuricemia (or SUA) and CAD is unclear.

## 2 Materials and methods

### 2.1 Study design and patient population

Since 1991, the data of patients undergoing coronary angiography (CAG) in Beijing Hospital have been recorded in the hospital database. In the present study, a retrospective analysis of this CAG registry was performed.

In total, 10,198 separate patients underwent CAG between September 1991 and February 2014. All participants were spoken with preoperatively and informed about the use of their records for research purposes. The local ethics committee waived the need for written informed consent because the data were analyzed anonymously for this retrospective cohort study based on the data stored in the hospital database. Of these patients, 5069 had a SUA level measured within 2 weeks before the day of CAG, and we excluded all patients receiving allopurinol, which reduced SUA levels. If patients had multiple SUA measurements within that period, the one closest to the time of CAG was used for this analysis. The patients were assessed before CAG, and a full clinical history was obtained, including information about age, sex, smoking habits, cardiovascular risk factors, and medical treatment. Body weight and height were measured and recorded for all patients, and body mass index (BMI) was calculated.

### 2.2 Definitions

Hyperuricemia was defined as >6 mg/dL (> 360 μmol/L) in women and >7 mg/dL (>420 μmol/L) in men. CAD was defined as the existence of stenosis ≥50% in diameter in any one of the coronary arteries or major branches, as detected by CAG. Diabetes was considered present if there was a documented diagnosis requiring treatment with medication or diet, and hypertension was considered present if there was a documented history of hypertension treated with medication. Hyperlipidemia was defined as a total cholesterol (TC) value >5.2 mmol/L or if the patient was receiving relevant drug therapy.

### 2.3 Statistical analysis

Continuous data are expressed as mean ± standard deviation or median (interquartile range), and categorical data as percentages. Analysis of variance (ANOVA) and nonparametric statistics such as the χ^2^ test were used for continuous and categorical variables, respectively. Linear-by-Linear Association was used for linear trend test. Multivariate logistic regression analysis was used to assess the effects of sex, age, smoking habits, hypertension, diabetes, hyperlipidemia, BMI, serum creatinine level, hyperuricemia, and any therapies that can affect SUA (including acetylsalicylic acid [ASA], angiotensin-receptor blockers [ARBs], and diuretics) on CAD. Statistical analysis was performed using the Statistical Package for Social Sciences (SPSS) for Windows (version 16; SPSS Inc., Chicago, IL), and a 2-tailed *P* < .05 was considered statistically significant.

## 3 Results

The baseline clinical characteristics of the study population are shown in Table 1. Of the 5069 patients who had their SUA levels measured before CAG, 3397 were men (mean age 62.0 ± 12.1 years) and 1672 were women (mean age 65.5 ± 10.4 years). CAD was present in 3433 patients (67.7%).

**Table 1:**
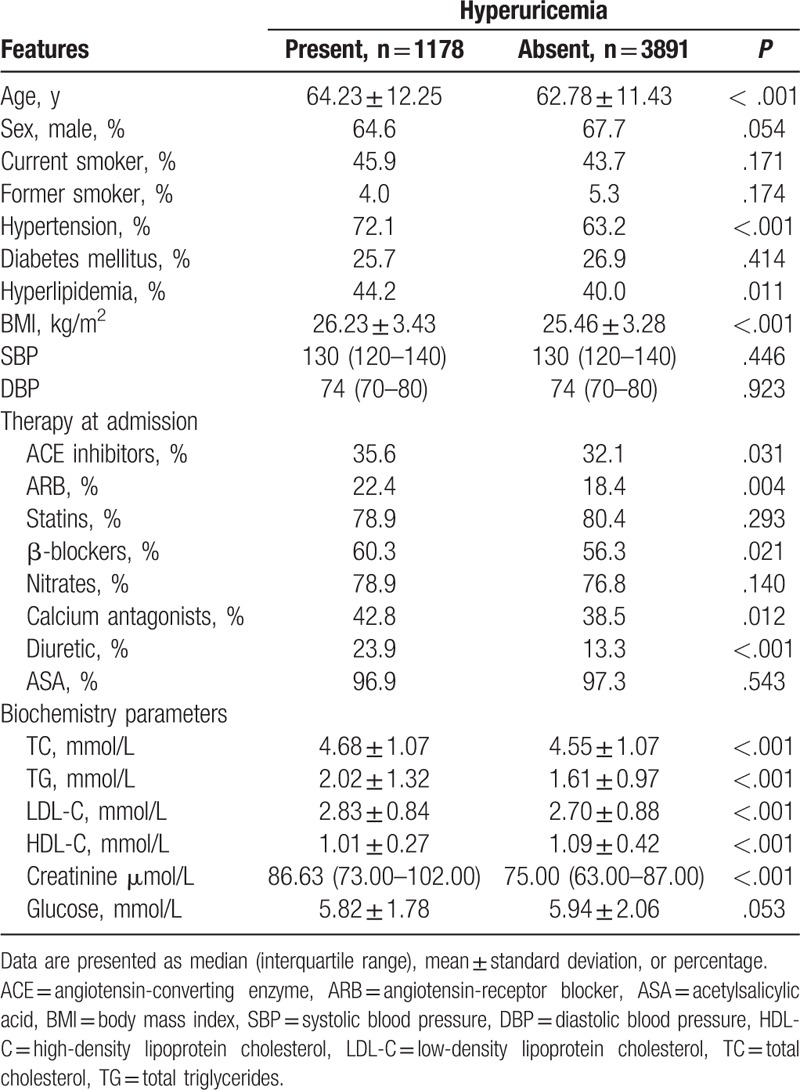
Patients’ baseline characteristics.

Patients were divided into 2 groups in terms of the presence or absence of hyperuricemia. There were significant differences between patients with or without hyperuricemia in mean age (64.23 ± 12.25 vs 62.78 ± 11.43 years, *P* < .001) and BMI (26.23 ± 3.43 vs 25.46 ± 3.28 kg/m^2^, *P* < .001). Patients with hyperuricemia were more likely to have a history of hypertension (72.1% vs 63.2%, *P* < .001) and hyperlipidemia (44.2% vs 40.0%, *P* = .011) than those without hyperuricemia. No difference in systolic blood pressure; diastolic blood pressure; and percentages of men, current smokers, former smokers, and those with diabetes mellitus was observed between the 2 groups. Patients with hyperuricemia were more likely at the time of admission to be on angiotensin-converting enzyme inhibitors (35.6% vs 32.1%, *P* = .031), ARBs (22.4% vs 18.4%, *P* = .004), β-blockers (60.3% vs 56.3%, *P* = .021), calcium antagonists (42.8% vs 38.5%, *P* = .012), and diuretics (23.9% vs 13.3%, *P* < .001). Significant differences were found between patients with or without hyperuricemia in terms of mean values of TC (4.68 ± 1.07 vs 4.55 ± 1.07 mmol/L, *P* < .001), triglycerides (2.02 ± 1.32 vs 1.61 ± .97 mmol/L, *P* < .001), low-density lipoprotein cholesterol (2.83 ± .84 vs 2.70 ± .88 mmol/L, *P* < .001), and high-density lipoprotein cholesterol (1.01 ± .27 vs 1.09 ± .42 mmol/L, *P* < .001); and median value of creatinine (86.63 [interquartile range: 73.00–102.00] μmol/L vs 75.00 [interquartile range: 63.00–87.00] μmol/L, *P* < .001) (Table 1).

Using the χ^2^ test, we found that the incidence of CAG was higher in the hyperuricemia group than in the nonhyperuricemia group (71.5% vs 66.6%, *P* = .002).

Multivariate logistic regression analysis was performed and adjusted for sex, age, smoking habits, hypertension, diabetes, hyperlipidemia, BMI, serum creatinine, ASA, ARBs, and diuretics. After multivariate logistic regression analysis, there was no independent correlation between hyperuricemia and CAD (odds ratio [OR] = 1.129, 95% confidence interval [CI], 0.941–1.355, *P* = .191) among all patients. However, there was an independent correlation between hyperuricemia and CAD for women (OR = 1.509, 95% CI, 1.106–2.057, *P* = .009) but not in men (OR = .881, 95% CI, 0.702–1.106, *P* = .274) (Table 2).

**Table 2:**
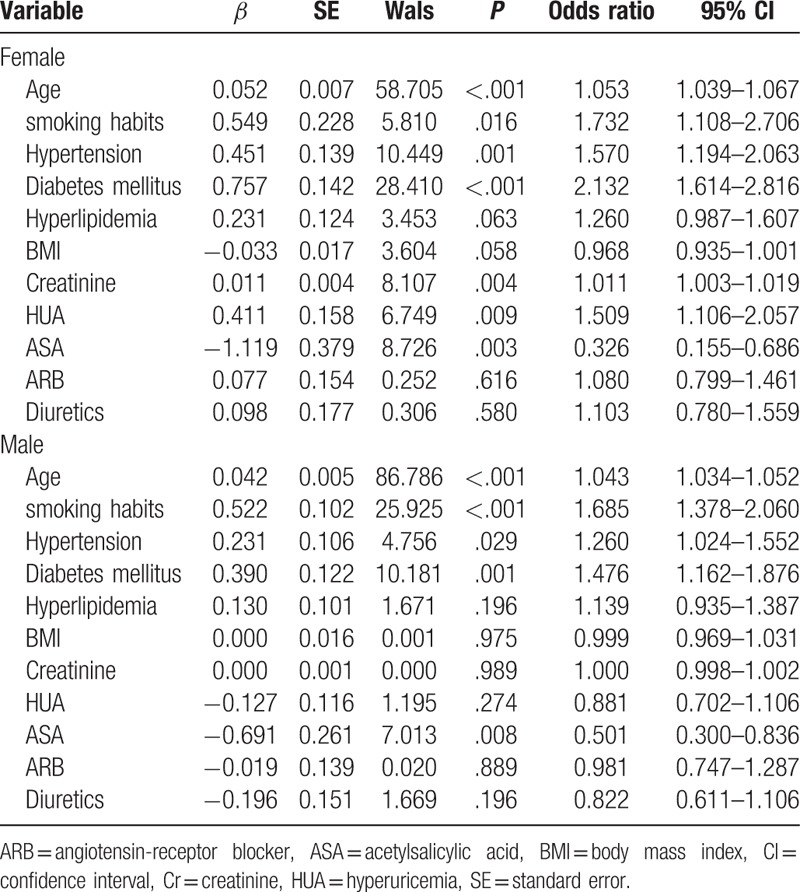
Results of logistic regression analysis of the effects of risk factors on coronary artery disease in female and male patients separately.

**Figure 1:**
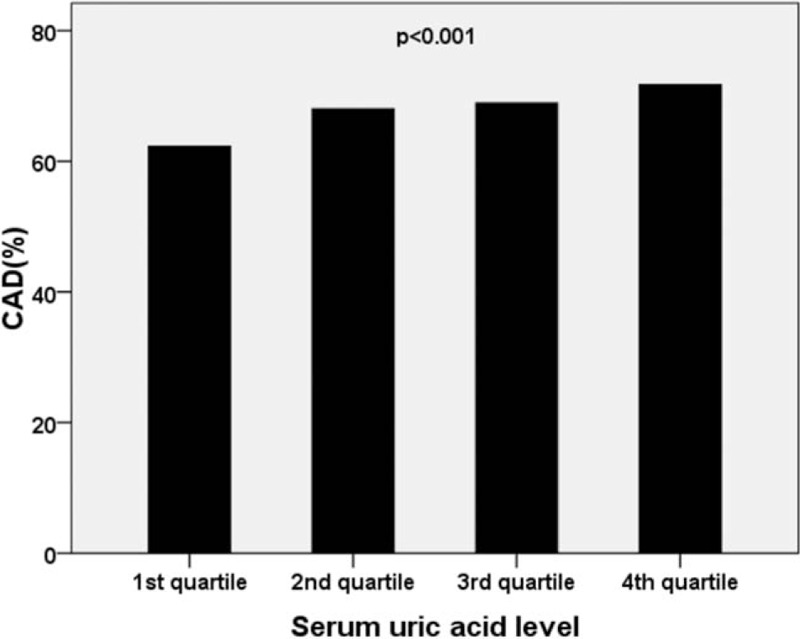
Prevalence of coronary artery disease (CAD) according to serum uric acid quartiles. After stratifying the patients into 4 groups according to their SUA quartiles, a trend was found that higher SUA quartiles were associated with higher percentage of CAD. The values were significantly different as a whole, and the incidence of CAD in quartile 1 was significantly different with any one of the other quartiles, but no significant difference was found among quartiles 2, 3, and 4.

When patients were stratified into 4 groups according to their SUA quartiles (the SUA levels were from 81.0 to 294.0 μmol/L, from 294.4 to 350.0 μmol/L, from 350.4 to 412.0 μmol/L, and from 412.2 to 897.0 μmol/L for quartiles 1, 2, 3, and 4, respectively in men, and the SUA levels were from 80.0 to 242.0 μmol/L, from 242.6 to 294.0 μmol/L, from 295.0 to 360.0 μmol/L, and from 360.6 to 715.0 μmol/L for quartiles 1, 2, 3, and 4, respectively in women), a significant association was found between the lower SUA quartiles and the prevalence and extent of CAD. There was a trend, higher SUA quartiles were associated with higher percentage of CAD (62.3%, 68.0%, 68.9%, and 71.7% for quartiles 1, 2, 3, and 4, respectively, *P* < .001) (Fig. 1). The values were significantly different as a whole, and we also found that the incidence of CAD in quartile 1 was significantly different with any one of the other quartiles, but no significant difference was found among quartiles 2, 3, and 4. In female group, this trend was found (49.3%, 55.4%, 60.0%, and 66.9% for quartiles 1, 2, 3, and 4, respectively, *P* < .001), and the incidence of CAD was significantly different among quartiles 1, 2, 3, and 4, except for quartiles 2 and 3. However, this trend was not found in male group (68.7%, 74.3%, 73.3%, and 74.1% for quartiles 1, 2, 3, and 4, respectively, *P* = .059).

**Figure 2:**
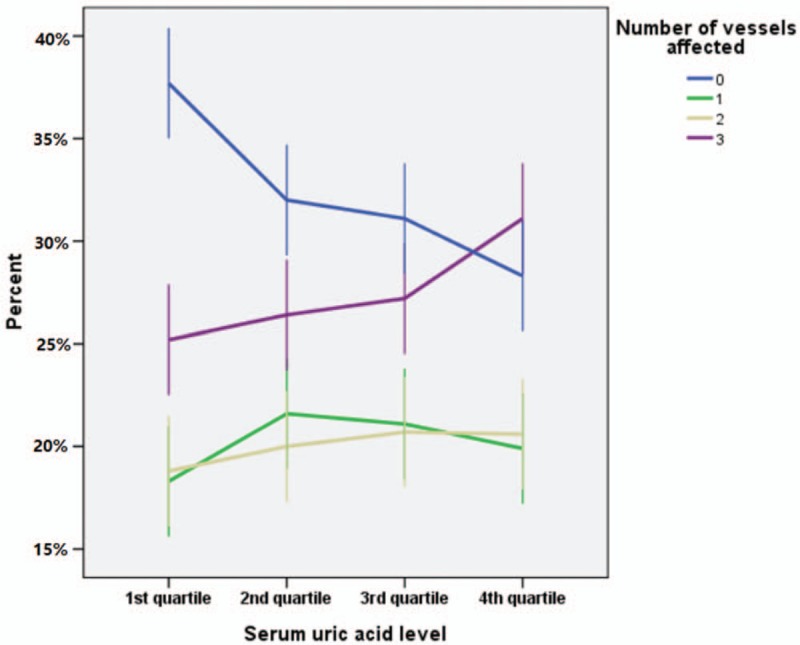
Number of vessels affected in different serum uric acid levels. The higher SUA quartiles were associated with higher incidence of 3-vessel disease, and the higher SUA quartiles were associated with lower incidence of normal vessel. And the incidence of 3-vessel in quartile 4 was significantly different as compared with those in other quartiles, but no significant difference was found among quartiles 1, 2, and 3; furthermore, the incidence of normal vessel in quartile 1 was significantly different with those of any other quartiles, and significant difference was also found between quartile 2 and 4, but no significant difference was found among other quartiles.

All patients were divided into 4 groups according to the number of diseased coronary arteries: normal,1-vessel disease, 2-vessel disease, or 3-vessel disease. The median number of diseased vessels in quartile 1 was 1 (interquartile range: 0–3), and that of quartiles 2, 3, and 4 was 2 (interquartile range: 1–3). There was a trend that the higher SUA quartiles were associated with higher incidence of 3-vessel disease (25.2%, 26.4%, 27.2%, and 31.1% for quartiles 1, 2, 3, and 4, respectively, *P* = .001), and the higher SUA quartiles were associated with lower incidence of normal vessel (37.7%, 32.0%, 31.1%, and 28.3% for quartiles 1, 2, 3, and 4, respectively, *P* < .001) (Fig. 2, error bars: 95% CI). This trend was not found in 1-vessel disease (18.3%, 21.6%, 21.1%, and 19.9% for quartiles 1, 2, 3, and 4, respectively, *P* = .407) or2-vessel disease (18.8%, 20.0%, 20.7%, and 20.6% for quartiles 1, 2, 3, and 4, respectively, *P* = .221). And the incidence of 3-vessel in quartile 4 was significantly different as compared with those in other quartiles, but no significant difference was found among quartiles 1, 2, and 3; furthermore, the incidence of normal vessel in quartile 1 was significantly different with those of any other quartiles, and significant difference was also found between quartile 2 and 4, but no significant difference was found among other quartiles. Moreover, we have analyzed the data in men and women separately and found a trend that the higher SUA quartiles were associated with higher incidence of 3-vessel disease (15.6%, 19.8%, 22.3%, and 27.6% for quartiles 1, 2, 3, and 4, respectively, *P* < .001), and the higher SUA quartiles were associated with lower incidence of normal vessel (50.7%, 44.6%, 40.0%, and 33.1% for quartiles 1, 2, 3, and 4, respectively, *P* < .001) in women. However, this trend was not found in men. We also found that, in women, the incidence of 3-vessel in quartile 4 was significantly different as compared with those in other quartiles, and significant difference was also found between quartiles 3 and 4, but no significant difference was found between quartiles 1 and 2, 2 and 3; the percentage of normal vessel disease was significantly different between any 2 of the quartiles 1, 2, 3, and 4, except for quartiles 2 and 3.

**Figure 3:**
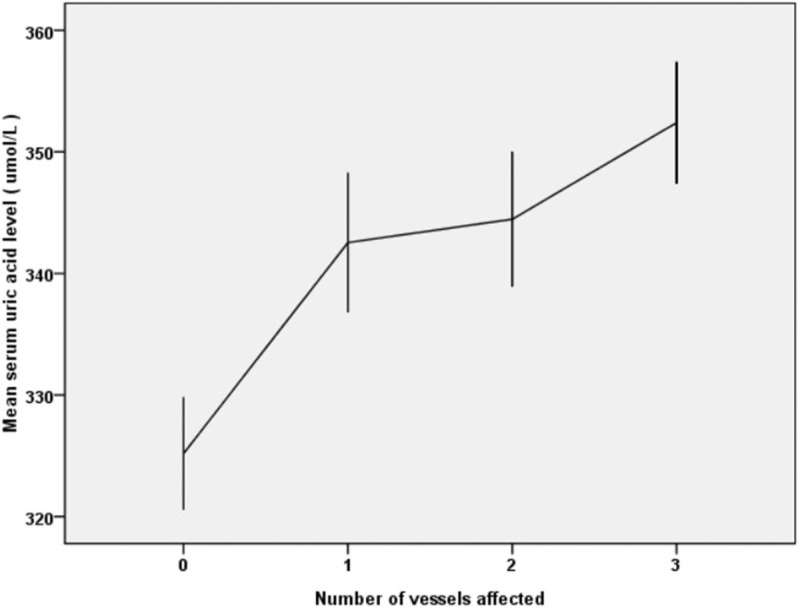
Mean serum uric acid level in groups with different numbers of vessels affected. The SUA level significantly increased as the number of affected vessels increased.

The SUA level increased significantly as the number of affected vessels increased, and there was a statistically significant difference between the groups by one-way ANOVA (*P* < .001; Fig. 3, error bars: 95% CI).

## 4 Discussion

The main findings of the present study are as follows: there was a larger number of patients with CAG in the hyperuricemia group than in the nonhyperuricemia group; after multivariate analysis, there was an independent correlation between hyperuricemia and CAD in women; there was a trend that higher SUA quartiles were associated with higher percentage of CAD in female group, but not in male group; there was another trend that patients in the higher SUA quartiles group had a higher incidence of 3-vessel disease than those in the lower SUA quartile groups, and after analyzing the data in men and women separately, we found this trend in women, but not in men; the SUA level significantly increased as the number of affected vessels increased.

SUA is the final oxidation product of purine catabolism in humans.^[11]^ It is formed from the breakdown of adenosine and guanine. Through the enzymatic action of xanthine oxidase (XO), the purine breakdown products, xanthine and hypoxanthine, are converted to SUA. During this reaction, molecular oxygen is reduced, and superoxide free radicals are generated. Thus, XO is responsible for forming 2 different molecules: SUA and free radicals. Oxygen free radicals are postulated to play a key role in vascular injury in cardiovascular and kidney disease.^[12]^ SUA acts like an antioxidant in the early stages of the atherosclerotic process and is the strongest determinant of plasma antioxidant capacity.^[13]^ When the SUA level increases to >6 mg/dL in women and 6.5 to 7.0 mg/dL in men, this antioxidant state is paradoxically reversed into a pro-oxidant state in the later stages of the atherosclerotic process.^[14,15]^ SUA has also been shown to have proliferative and proinflammatory effects on vascular smooth muscle, which can lead to hypertension and vascular disease. Finally, SUA has been also found to upregulate C-reactive protein, an active component in the inflammatory process, in human vascular cells and endothelial cells, thereby providing another potential mechanism by which SUA is directly involved in the proinflammatory and proatherogenic process.^[16–18]^

Should hyperuricemia be considered a cardiovascular risk factor? This question is a matter of controversy. Several epidemiologic studies.^[2,4,10,19]^ have suggested that there is an association between hyperuricemia and CAD. In our study, the incidence of CAG was higher in the hyperuricemia group than in the nonhyperuricemia group. Nevertheless, hyperuricemia is often associated with established cardiovascular risk factors. There were significant differences in mean age, BMI, and percentages of those with hypertension and hyperlipidemia between patients with or without hyperuricemia in our study (Table 1), and after multivariate logistic regression analysis, there was no independent correlation between hyperuricemia and CAD. This observation is supported by several studies,^[3,20,21]^ such as the study by De Luca et al who reported no significant relationship between SUA and the prevalence of CAD after correction for baseline confounding factors.

However, our study did show an independent correlation between hyperuricemia and CAD for female patients after multivariate analysis, and the higher SUA quartiles were associated with higher incidence of 3-vessel disease in women. Our finding is supported by the following results of several other studies. In the study by Culleton et al,^[22]^ conducted in 6763 individuals from the Framingham Heart Study, an increased risk for adverse outcome after age adjustment was found for women only. In the First National Health and Nutrition Examination Survey (NHANES I) epidemiologic follow-up studies,^[23]^ there was no association between hyperuricemia and CAD for men, but there was an association for women; additionally, SUA levels were predictive of mortality from all causes and from ischemic heart disease in women. In a study on a Belgian population,^[24]^ the data showed that the SUA level was associated with total, cardiovascular, and coronary mortality in women only. In a study from Chicago,^[25]^ the SUA level was associated with abnormal electrocardiograms and total mortality in women only. In the study by Kim et al,^[19]^ there was no significant association between hyperuricemia and CAD incidence or mortality in men, but there was an increased risk for CAD mortality in women (OR = 1.67; 95% CI, 1.30–2.04).

In light of the association between hyperuricemia and insulin resistance, the sex-specific difference in the association of SUA with CAD risk observed in our study may relate to a sex-specific difference in the effect of diabetes on CAD. In particular, diabetes has been found to confer a greater risk for CAD in women than in men. ^[26]^ In the NHANES analysis, ^[22]^ SUA was higher in women with diabetes than in women without, but no such difference was found among men with or without diabetes.

The present study showed that the prevalence of CAD increased across all SUA level quartiles, and the SUA level increased with the number of diseased vessels. These results are supported by several studies that have investigated the relationship between SUA and the presence of CAD.^[27–30]^ However, few studies have addressed the relationship between the SUA level and severity of CAD. In our study, the incidence of 3-vessel disease was higher in patients belonging to the higher SUA quartile groups than in those belonging to the lower SUA quartile groups. Duran et al ^[31]^ reported that patients with hyperuricemia had a larger number of diseased vessels, that the SUA level was significantly associated with the number of diseased vessels, and that SUA was an independent risk factor for multi-vessel disease. Ehsan Qureshi et al^[32]^ also reported that hyperuricemia was associated with higher numbers of total occlusions and critical lesions in men presenting with acute coronary syndrome. In another study, Spoon et al ^[33]^ demonstrated that the group with hyperuricemia had a higher incidence of 3-vessel disease than did the group without elevated SUA levels. However, the populations in these 3 studies were smaller than that in our study.

The present study has some potential limitations. First, the study population consisted of patients referred to our hospital; therefore, it does not fully represent the general population. Thus, it might contain selection biases. Second, we could not control exposure or outcome assessment; instead, we must rely on our hospital database and documented histories. When information comes from patients’ recall, some documented histories might be inaccurate and subject to biases. In addition, we assessed the severity of coronary atherosclerosis by the number of diseased vessels. The SYNTAX score and Gensini score provide more detailed information about CAD rather than the number of diseased vessels. Finally, patients’ genetic background was not considered in our study. Christopher et al^[34]^ reported that there was a significant association between carriage of the PlA2 allele and CAD for younger subjects. Iaccarino et al^[35]^ reported that there was a close relationship between calmodulin-dependent protein kinase 4 and endothelial nitric oxide synthase in the endothelium, which might result in endothelial dysfunction.

Our study has several important strengths. Importantly, our research included a large number of patients, which helped increase the precision of estimates while minimizing heterogeneity. Moreover, we performed sex-specific subgroup analyses of this study fully adjusting for traditional risk factors for CAD.

## 5 Conclusions

We found an independent association between hyperuricemia and the presence of CAD in women, and there was a trend, that a higher SUA level was associated with a higher incidence of 3-vessel disease, and after analyzing the data in men and women separately, we found this trend in women, but not in men. In addition to the evaluation of conventional risk factors in daily clinical practice, measurement of the SUA level might provide significant prognostic benefits in terms of global cardiovascular risk and management of patients; further, high levels of SUA may become surrogate markers of CAD severity, especially in women.

## Acknowledgments

We thank the field workers for their contribution.

## Author contributions

**Conceptualization:** Ming Lan.

**Data curation:** Ming Lan, Bing Liu.

**Formal analysis:** Ming Lan.

**Methodology:** Ming Lan.

**Project administration:** Ming Lan, Bing Liu.

**Resources:** Ming Lan.

**Software:** Ming Lan.

**Supervision:** Qing he.

**Visualization:** Ming LAN.

**Writing–original draft:** Ming LAN.

**Writing–review and editing:** Ming LAN, Qing HE.
